# Volumetric analysis of central body fat accurately predicts incidence of diabetes and hypertension in adults

**DOI:** 10.1186/s40608-015-0039-3

**Published:** 2015-02-25

**Authors:** Jacob T Gibby, Dennis K Njeru, Steven T Cvetko, Ray M Merrill, Benjamin T Bikman, Wendell A Gibby

**Affiliations:** Departments of Physiology and Developmental Biology, Provo, UT USA; Health Science, Brigham Young University, Provo, UT USA; Accuscan Health Imaging Center, Salt Lake City, UT 84101 USA; Novarad, American Fork, UT 84003 USA

**Keywords:** Volumetric Analysis, CT Scan, Obesity, Diabetes, Hypertension

## Abstract

**Background:**

Central adipose tissue is appreciated as a risk factor for cardiometabolic disorders. The purpose of this study was to determine the efficacy of a volumetric 3D analysis of central adipose tissue in predicting disease.

Full body computerized tomography (CT) scans were obtained from 1225 female (518) and male (707) subjects, aged 18–88. Percent central body fat (%cBF) was determined by quantifying the adipose tissue volume from the dome of the liver to the pubic symphysis. Calcium score was determined from the calcium content of coronary arteries. Relationships between %cBF, BMI, and several cardiometabolic disorders were assessed controlling for age, sex, and race.

**Results:**

Higher %cBF was significantly greater for those with type 2 diabetes and hypertension, but not stroke or hypercholesterolemia. Simple anthropometric determination of BMI equally correlated with diabetes and hypertension as central body fat. Calcium scoring significantly correlated with all measurements of cardiovascular health, including hypertension, hypercholesterolemia, and heart disease.

**Conclusions:**

Central body fat and BMI equally and highly predict incidence of hypertension and type 2 diabetes.

## Background

The consistent and robust increased risk of multiple diseases associated with body fat has led to greater appreciation of adipose tissue analysis. Excess body fat increases risk of certain cancers [1], diabetes [2], hypertension [2], and cognitive dysfunction [3]. Considering the substantial economic and personal burden of these diseases, and the increasing worldwide trends of obesity [4,5], the ability to detect and ultimately mitigate weight gain is paramount. The majority of research has focused on whole body fat, employing various classic (e.g., Body Mass Index (BMI), underwater weighing [6]) and modern (e.g., dual-energy X-ray absorptiometry [7]) methods. However, research in recent decades has benefitted from identifying distinct fat depots by again using both simple (e.g., waist circumference, waist-to-hip ratio [8]) and complex (e.g., magnetic resonance imaging (MRI) [9]) techniques. Among the multiple sites of fat storage on the body, the greatest advance has been the ability to quantify internal fat depots, in particular central fat, via MRI [9] and computed tomography [10], which is important given the evidence suggesting the pathological role of central fat in various disease states [11]. Thus, improved methods of quantifying central fat may be useful in determining risk of related diseases.

Despite its acceptance and validation [12], there is a broad range of analytical methodologies in determining abdominal adiposity via computerized tomography (CT) imaging that has complicated its widespread application. The common practice is to extrapolate central body fat amount through a single CT slice [13,14]. The purpose of this study was to validate the use of assessing central body fat via CT with regards to cardiometabolic disease risk. To this end, we employed a novel volumetric analysis to quantify abdominal fat in men and women and measured its correlation with several diseases associated with excess central adipose storage. Given the widespread acceptance of coronary artery calcium scoring in assessing cardiovascular disease [15], we also included calcium scoring to determine its ability to predict patients with cardiometabolic diseases.

## Methods

### Patients

Analyses are based on 1225 patients who received full body CT scans using a 4-slice multi-detector GE Lightspeed CT scanner (Milwaukee, Wisconsin) at Accuscan Health Imaging Center. Histories and clinical information of patients were prepared and given by Accuscan Health Imaging Center. All subjects were 18 years of age or older. Both pre and postmenopausal women were included in the study. The majority of patients were Caucasian. The data were collected from patients receiving routine check-up CT scans and providing informed consent. The study was approved by the Brigham Young University Institutional Review Board.

### Clinical information

Clinical information was self-reported by answering a questionnaire. Hypertension was defined as having a history of hypertension for at least one year (i.e., blood pressure > 140/90 mmHg). Diabetes was defined as a patient clinically diagnosed with type 2 diabetes mellitus (i.e., blood glucose >126 mg/dl) and requiring insulin or oral hypoglycemic drugs. Heart disease was defined as a patient having had a heart attack, coronary bypass, heart stent, or percutaneous transluminal coronary angioplasty (PTCA). High Cholesterol was defined as total cholesterol above 200 mg/dL or requiring lipid-lowering drugs. Stroke was defined as a patient having had a stroke in their lifetime. Cancer was defined as a patient having been clinically diagnosed with cancer in their lifetime. Body mass index was calculated as body mass (kg) divided by height^2^ (m) [16].

### Scan parameters

All subjects were scanned in the supine position. The tube voltage was set at 140 kV, and the current varied between 100–360 mA (5-95th percentile) depending on the weight of the patient. Each subject was scanned in a large field view with 5 mm thick scans, with a 5 mm interval. Reconstructive images were created every 2.5 mm to enhance detail and limit noise. The CT scanner was calibrated each day with standard phantoms. Water was set to a density of 0 according to the Hounsfield Scale [17].

### Hounsfield scale

An algorithm was designed to measure pixel intensity in CT scans correlated to types of body tissue. The pixel intensities used to distinguish various tissues were derived from their corresponding intensities, which were computed using the Hounsfield scale. The Hounsfield scale relates relative densities given in Hounsfield Units (HU); water is equal to 0, air is equal to −1000, bone is approximately +700 to +3000, and fat is approximately −100 to −50 [17]. In measuring percent central body fat (%cBF), we used a range of −150 HU to −15 HU to represent adipose tissue. We neglected the range of −15 HU to 0 HU. Body range was set at −150 HU to +3000 HU.

A notable challenge is defining a pixel containing tissues of disparate densities [18]. This is common in the lumen of the digestive tract and dermis. False-positive fat pixels would arise on the boundary between air and higher-density tissues. To test the false positive volume percentage, we analyzed all regions containing air by one pixel and count the number of pixels also labeled as adipose tissue. In analyzing scans from 10 randomly selected patient samples (via random number generation), we determined a mean, false-positive rate of 2.64%. While this is a potentially significant percentage, the standard deviation was 0.44%, indicating that nearly all patients had relatively the same percent false positive.

### Calcium scoring

Chest CT scans were used by a certified technician to determine calcium scoring. Each scan was implemented in the workstation interface for the CT scanner previously mentioned. Coronary artery calcium score was assessed according to a predefined phantom standard [19,20]. The Agatston score was then used to determine and scale calcium scoring [21].

### Percent central body fat measurement

Percent central body fat (%cBF) is an in-house term to indicate the percentage of adipose tissue from the dome of the liver to the pubic symphysis. This includes the intra-abdominal, retroperitoneal, mesenteric, and omental fat regions, as described [22]. Percent central body fat was determined using a Novarad® NovaPACS (Picture Archiving and Computing System) 3D workstation. Our algorithm is applied to a 3D CT dataset. The user can narrow the region of interest by adjusting 6 sides of an enclosed box. To standardize the location of measurements, we set bounds of the CT 3D rendering from the dome of the liver to the bottom of the pubic symphysis. All other dimensions were extended to include the entire abdomen. The tissues are presented as volume in liters (L) by:

$$ Pixel\  volume = Pixel\  Row\  Spacing\ *\  Pixel\  Column\  Spacing\ *\  Pixel\  Slice\  Spacing $$

$$ Tissue\  volume = Tissue\  Pixel\  Count\ *\  Pixel\  Volume $$

The percentage body tissue is thus computed by:

$$ \% Tissue = \left(\sum tissue\  pixels\ /\sum body\  pixels\right)\ *\ 100 $$

**Figure 1:**
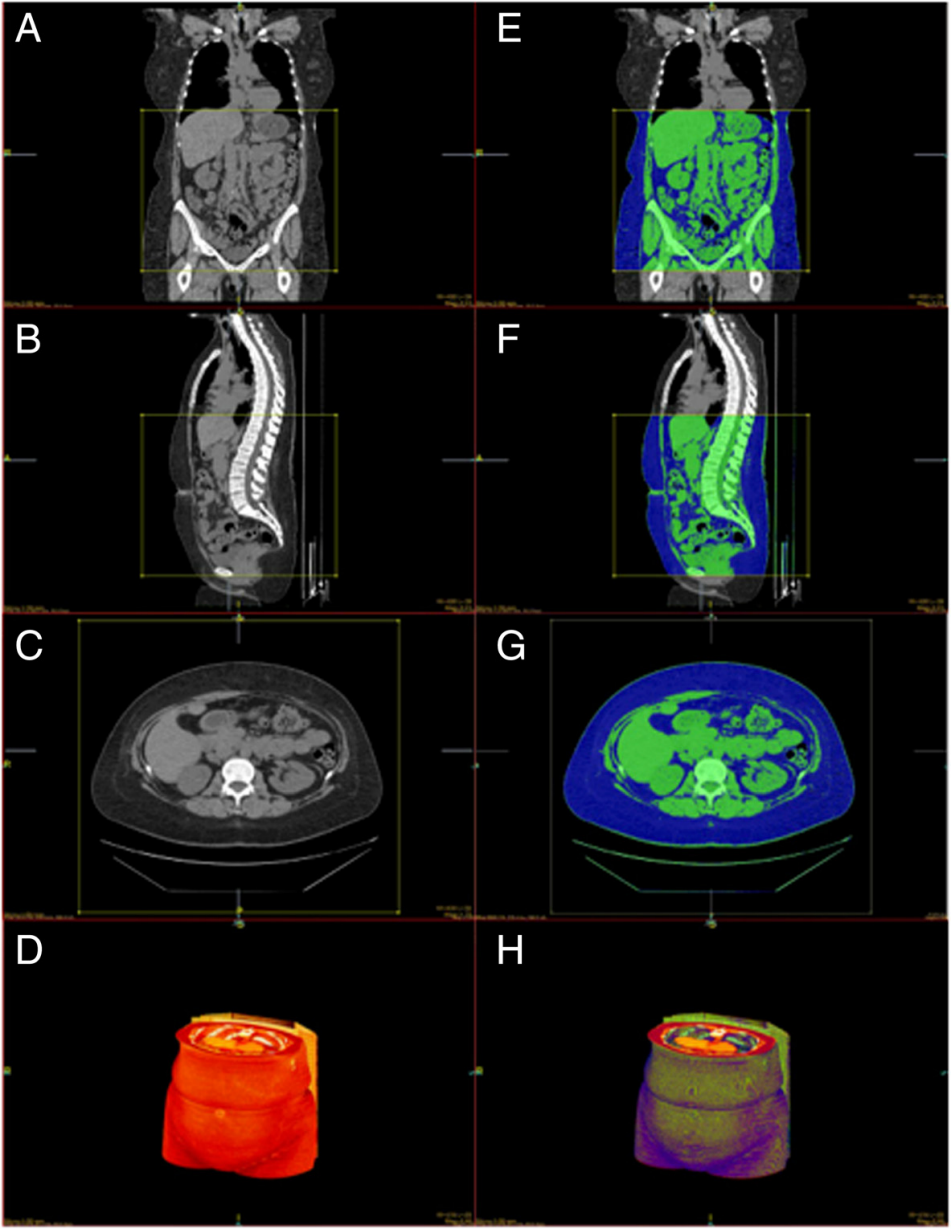
**A typical 2D snapshot of the 3D rendering from a CT scan using NovaPACS.** On the left, normal coronal **(A)**, sagittal **(B)**, axial **(C)**, and 3D rendering **(D)** images are displayed. On the right, %cBF has been calculated in our set bounds with adipose tissue depicted in blue, all other body tissue depicted in green, and air depicted in black. Coronal **(E)**, sagittal **(F)**, axial **(G)**, and 3D rendering **(H)** images are depicted.

The %cBF of each subject was estimated. The software automatically excludes any pixels containing air in lung, bowel lumen, or exterior to the body. A typical 2D snapshot of the 3D rendering software used on the NovaPACS system is shown in Figure 1.

### Statistical techniques

Frequencies, means, and standard deviations were used to describe the data. Regression analysis was used to assess the association between BMI and selected biometric variables and current and former smoking status, adjusting for age, sex, and race. The race variable represented 1164 (95.02%) Caucasians and 61 (4.98%) other. The other category consisted of 12 Asians, 10 Hispanics, 9 of other race, and 30 with unknown race. Regression analysis was also used to assess the association between %cBF and selected biometric variables and current and former smoking status, adjusting for age, sex, and race. The probability of diabetes given the subject had hypertension was compared with the probability of diabetes given the subject did not have hypertension. The ability for BMI to predict diabetes and hypertension was evaluated using different cut points for BMI. True positive (sensitivity) was compared with false positives (1-specificity) at each cut point and graphed using a receiver operating characteristic (ROC) curve. This was also done with different cut points for percent body fat. Two-sided tests of significance were based on the 0.05 level against a null hypothesis of no association. Analyses were performed using SAS version 9.3 (SAS Institute Inc., Cary, NC, USA, 2010).

## Results

Study subjects ranged in age from 18 to 88 (M = 53.4 y, SD = 10.5). Body Mass Index (BMI) ranged from 16.7 to 53.2 (M = 27.5 kg/m^2^, SD = 4.9) and percent central body fat (%cBF) ranged from 13.9 to 73.2 (M = 48.3%, SD = 9.3). The majority of subjects were men (707, 57.7%) compared with women (518, 42.3%) and Caucasian (1164, 95.0%). A description of selected health measures and smoking behavior is presented in Table 1. Hypertension and high cholesterol were the most common health problems, followed by diabetes and then cancer; heart disease and stroke were least common. The table also shows mean BMI, %cBF, and calcium score by the selected health measures and smoking behavior. All three mean scores were significantly greater among those with hypertension and former smokers. Mean BMI and %cBF were significantly greater for those with diabetes and hypertension. Mean calcium score was the only of the three outcome measures to distinguish heart disease.

**Table 1:** **Disease and smoking status according to mean BMI, percent central body fat, and calcium score**

**Status**	**No. of subjects**	**% of total subjects**	**BMI** * mean	**P value** ^**†**^	**% cBF** * mean	**P value** ^**†**^	**Calcium** * score mean	**P value** ^**†**^
**Hypertension**								
Yes	222	18.1	29.3	<0.0001	50.8	<0.0001	205.8	0.0001
No	1003	81.9	27.1		47.8		87.0	
**Diabetes**								
Yes	88	7.2	31.0	<0.0001	52.1	<0.0001	159.2	0.2085
No	1137	92.8	27.3		48.0		102.2	
**Heart disease**								
Yes	37	3.0	27.7	0.7911	48.6	0.8232	539.0	<0.0001
No	1188	97.0	27.5		48.3		95.0	
**High cholesterol**								
Yes	240	19.6	27.8	0.3600	48.8	0.3342	199.3	0.0001
No	984	80.4	27.5		48.2		84.1	
**Stroke**								
Yes	6	0.5	25.7	0.3587	48.0	0.9318	0	0.5155
No	1219	99.5	27.5		48.3		105.8	
**Cancer**								
Yes	54	4.4	28.0	0.4760	48.5	0.8708	129.4	0.6632
No	1171	95.6	27.5		48.3		104.6	
**Current smoker**								
Yes	85	6.9	26.0	0.0608	47.6	0.1586	111.1	0.8949
No	1140	93.1	27.0		48.9		105.0	
**Former smoker**								
Yes	220	18.0	27.6	0.0250	50.0	0.0215	162.6	0.0175
No	1005	82.0	26.8		48.5		90.4	

*Adjusting for age, sex, and race. ^†^From the F statistic based on type III sums of squares.

**Figure 2:**
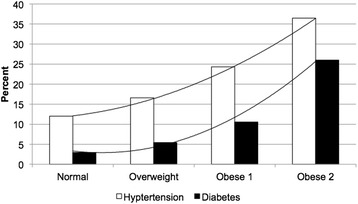
**Percent of subjects with hypertension or diabetes by BMI classification.** Normal: 18–24; Overweight: 25–29; Obese 1: 30–34; Obese 2: 35 + .

**Figure 3:**
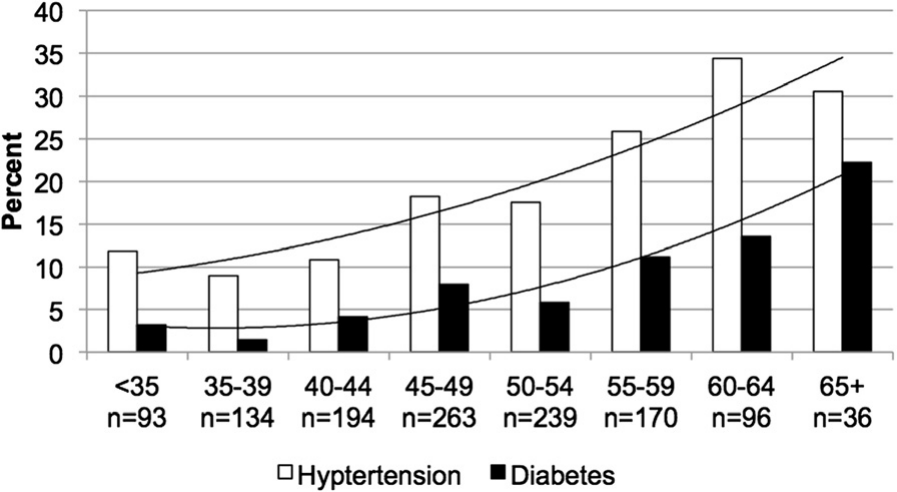
**Percent of subjects with hypertension or diabetes by percent central fat classification.**

Overall, mean BMI was 27.5 kg/m^2^ (SD = 4.9), mean %cBF was 48.3% (SD = 0.3), and mean calcium score was 137.2 (SD = 432). Body mass index and %cBF were strongly correlated (Pearson correlation coefficient = 0.61, p < 0.0001). Calcium score was very slightly positively correlated with BMI (Pearson correlation coefficient = 0.08, p = 0.0073), but not with %cBF (p = 0.1165). Regression analysis was performed for those variables significantly associated with BMI and %cBF, adjusting for age, sex, and race (Table 2). In particular, our data indicate individuals classified as overweight or obese based on BMI have a two- and threefold increased likelihood of hypertension compared with those of normal weight, respectively. The percent of subjects with hypertension or diabetes is shown by BMI classification (Figure 2) and %cBF grouping (Figure 3). Hence, both hypertension and diabetes similarly show a strong positive association with BMI and %cBF.

**Table 2:** **Body mass index, percent central body fat and calcium scores according to selected variables**

	**BMI**			**%cBF**			**Calcium Score**		
	Slope estimate*	Standard error*	**P-value** ^**†**^	Slope estimate*	Standard error*	**P-value** ^**†**^	Slope estimate*	Standard error*	**P-value** ^**†**^
Hypertension	2.14	0.36	<0.0001	3.06	0.64	<0.0001	−118.9	30.73	0.0001
Heart disease	−0.21	0.81	0.7911	−0.32	1.43	0.8232	−444.1	68.34	< 0.0001
High cholesterol	−0.32	0.35	0.3600	−0.61	0.63	0.3342	−115.3	29.93	0.0001
Diabetes	3.73	0.53	<0.0001	4.09	0.95	<0.0001	−57.0	45.26	0.2085
Former smoker	0.8	0.36	0.0250	1.47	0.64	0.0215	−72.2	30.34	0.0175

*Adjusting for age, sex, and race. ^†^From the F statistic based on type III sums of squares. Fifteen different models are represented in the table.

Each of the adjusted models represented in Table 2 were further assessed for significant interaction terms involving each of the main independent variables (i.e., hypertension, heart disease, high cholesterol, diabetes, and former smoker) and age, sex and race. Interaction terms involving sex and race were not significant in any of the 15 models. On the other hand, two interaction terms involving age were significant. In the model with BMI and former smoking status (yes vs. no), a significant interaction was present (p = 0.0460) involving former smoking and age. In the model with %cBF and former smoking status, a significant interaction was also present (P = 0.0020) between former smoking and age. The age variable is normally distributed, with both the mean and median equal to 53. Using age 53 as the cut point, the following table shows that the association between former smoking and BMI or %cBF is positive for ages < 53, but no association exists for ages 53+ (Table 3).

**Table 3:** **Body mass index and percent central body fat according to former smoking status**

**BMI correlations**	**Slope estimate**	**Standard error**	**P-value**	**%BF correlations**	**Slope estimate**	**Standard error**	**P-value**
<53	1.56	0.55	0.0048	<53	3.38	1.06	0.0015
53+	0.31	0.48	0.5223	53+	0.25	0.29	0.7702

**Figure 4:**
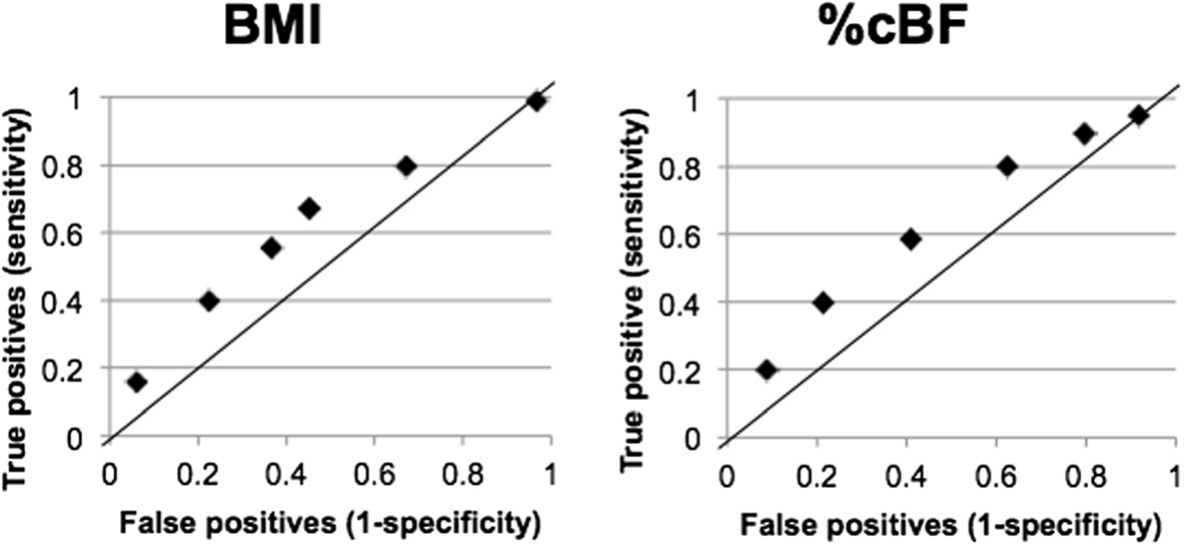
**ROC curves for hypertension.**

**Figure 5:**
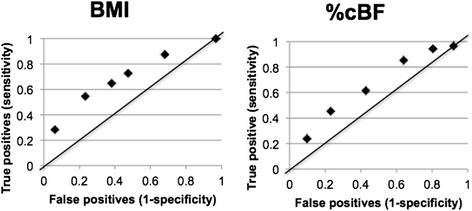
**ROC curves for diabetes.**

Receiver Operating Characteristic (ROC) curves were derived for different BMI cut points and different %cBF cut points. Figure 4 shows the ROC curve for hypertension and Figure 5 shows the ROC curve for diabetes. The line connecting the 0,0 point to the 1,1 point on the graphs represents where there is no predictive value in the test for hypertension or diabetes. Cut points reflected by dots in the direction of the 0,1 point on the graphs are predictive, with those closer to this upper-left hand corner of the graph superior. A BMI cut point of 28 and a %cBF of 45 does the best job detecting hypertension. A BMI cut point of 30 and a %cBF of 55 does the best job detecting diabetes.

## Discussion

The purpose of this study was to determine the efficacy of a novel and highly sensitive method of determining central adipose in predicting chronic disease. We found that %cBF significantly correlated with type 2 diabetes mellitus and hypertension, but not other selected chronic cardiometabolic diseases, such as stroke or hypercholesterolemia. Body mass index was similarly correlated with the same two diseases (i.e., diabetes and hypertension), and was at least as effective at predicting incidence of the diseases as our assessment of abdominal adiposity.

Heart disease is the leading cause of death in the U.S. [23]. Of the myriad factors that increase risk of heart disease, we measured prevalence of the four main modifiable factors, namely hypertension, smoking status, dyslipidemia, and diabetes [24], through questionnaire. While actual incidence of heart disease was not correlated with %cBF in our study, we did observe a significant correlation with hypertension, diabetes, and former smoking status. The lack of a correlation with heart disease may be a function of age; age is considered the most critical risk factor [25] and our patients are younger than patients in several similar studies. Another important distinction is our method; whereas the majority of published results use a single-slice scan of the abdomen to assess central fat, our method includes a multiple scan 3D rendering, allowing conclusive volumetric determination of central fat mass. These differences in analysis may explain disparate results from previous work [26].

Due to the high presence of calcium in atherosclerotic plaque, calcium scoring is accurate in predicting incidence of cardiovascular burden [27]. Our results corroborate previous work [28]; we found that calcium score accurately distinguishes hypertension, high cholesterol, heart disease, and former smoking. It is consistent with %cBF and BMI in distinguishing hypertension and former smoking. However, unlike %cBF and BMI, calcium score does not adequately distinguish diabetes.

Our findings of a consistent and significant relation between percent central fat and diabetes and hypertension are not surprising—these two variables are consistently observed together [29] with clear and common etiologies. The overwhelming majority of evidence supports a causal relationship wherein diabetes precedes the development of hypertension [29], though there is limited evidence to the opposite [30]. A critical mediator that links diabetes and hypertension is insulin resistance. Indeed, insulin resistance, which is the key pathological event in the development of diabetes, mediates multiple pathological processes in the development of hypertension, including Na^+^ retention, sympathetic nervous system activation, proliferation of vascular smooth muscle cells, and loss of nitric oxide-induced vasodilation [29,31]. Moreover, blood pressure improves with insulin-sensitizing medication [32]. However, insulin resistance is often undiagnosed due to the possibility of an insulin-resistant patient to have normal blood glucose. Thus, a potential utility of quantifying central body fat may be in detecting a patient who is at risk of developing or already has insulin resistance. This will require further studies to validate.

Cigarette smoking is recognized as a cause of insulin resistance [33]. Given the mediating role of insulin resistance in a host of cardiometabolic diseases, this observation indicates that insulin resistance may be the mediating mechanism whereby smoking results in dyslipidemia [31], hypertension [31,34], atherosclerosis [31,34], and hepatic steatosis [35]. This is consistent with current smokers being more likely to suffer hypertension than non-smokers. The lack of evidence linking smoking and diabetes may be a consequence of sample size in the current study. However, it may also be that by relying on diagnosis of diabetes, which is based solely on blood glucose values above 126 mg/dL, we may be missing patients who are pre-diabetic. Also, while smokers are generally considered to be leaner than nonsmokers, BMI and visceral fat were similar between smokers and nonsmokers. However, in corroboration with other reports [36], former smokers over 55 years of age had higher BMI and central body fat.

A weakness is the inherent self-selection of this study; subjects voluntarily came to the clinic for a check-up. Moreover, our assessment of disease was based on self-reported data, not actual in-clinic measurements (e.g., cholesterol, blood pressure). Moreover, our subject population is almost entirely Caucasian. This is noteworthy considering that ethnicity carries not only an inherent disparate propensity towards central adiposity [37], but also likelihood of developing subsequent disease [38]. Thus, our findings should be interpreted with caution when applied to diverse ethnicities.

## Conclusion

In conclusion, a main motive for this study was to determine whether quantification of central body fat might reveal a novel predictive diagnostic for chronic diseases. In light of our findings, analysis of central body fat may serve as an early marker of future disease onset. In other words, a patient with increased central body fat, but neither diabetes nor hypertension currently, may be warned of an increased disease risk in the absence of lifestyle changes. Our findings of similar predictive power with measuring percent central body fat and BMI are noteworthy. While much discussion has centered on the importance of central body fat, and we agree with this sentiment, it is important for physicians to bear in mind the efficacy of BMI to predict diabetes and hypertension. Despite the inherent flaw of BMI to ‘misdiagnose’ overweight or obesity in lean, muscled individuals, BMI can be used as a surprisingly accurate marker of visceral fat [39]. In particular, our data indicate individuals classified as overweight or obese based on BMI have a two- and threefold increased likelihood of hypertension compared with normal weight, respectively. The likelihood of developing diabetes is even greater in the overweight and obese, four- and nine-fold higher, respectively. In the end, these data add to the chorus of warning against the dangers of excess central adipose and validate the utility of simple anthropometry in determining disease risk.
